# Successful Decompression and Reduction of Displaced Sphenoid Wing Fracture into the Superior Orbital Fissure Causing Complete Ophthalmoplegia Secondary to Compression of Cranial Nerves III, IV, and VI

**DOI:** 10.1055/a-2780-4305

**Published:** 2026-05-11

**Authors:** Alex Tran, David Straughan, Davide M. Croci

**Affiliations:** 1 Department of General Surgery 719936 Lakeland Regional Health Systems IncLakelandFloridaUnited States; 2 Department of Plastic and Reconstructive SurgeryLakeland Regional HealthLakelandFloridaUnited States; 3 Department of Neurosurgery 33697 USF Health Morsani College of MedicineTampaFloridaUnited States

**Keywords:** ophthalmoplegia, sphenoid wing fracture, superior orbital fissure syndrome, ranial nerve compression

## Abstract

Superior orbital fissure syndrome (SOFS) is a rare, vision-threatening condition caused by injury or compression of cranial nerves III, IV, and VI. We report a 16-year-old male who sustained extensive craniofacial trauma, including a displaced left sphenoid wing fracture, after a motorized scooter accident. Initially, he had limited extraocular motility, but by hospital day 5, he developed complete ophthalmoplegia due to progressive nerve compression. Urgent surgical decompression of the superior orbital fissure and reduction of the fracture were performed via a pterional craniotomy on day 7. Postoperatively, the patient experienced full restoration of ocular motility and pupillary function, with no complications. This case highlights the potential for delayed neurological deterioration in sphenoid wing fractures and emphasizes the importance of close serial examination and timely operative intervention once SOFS develops, demonstrating that even delayed intervention can result in complete neurological recovery.

## Introduction

Superior orbital fissure syndrome (SOFS) is a rare, but severe condition characterized by the involvement of cranial nerves passing through the superior orbital fissure (SOF), including cranial nerves III (oculomotor), IV (trochlear), V1 (ophthalmic branch of the trigeminal nerve), and VI (abducens). 1 These nerves, along with the superior ophthalmic vein, traverse the SOF, a key anatomical structure located between the greater and lesser wings of the sphenoid bone. Traumatic-induced SOFS is uncommon, but when present, it may result in ophthalmoplegia, ptosis, pupillary dysfunction, sensory loss, or vision impairment. Early recognition and appropriate intervention are essential to prevent permanent neurological deficit.

Trauma-induced SOFS is uncommon and typically results from high-energy impact injuries, such as motor vehicle accidents, falls, or blunt force trauma to the orbit and surrounding cranial structures. The syndrome manifests with a range of clinical symptoms depending on the extent of nerve involvement and the severity of the fracture. Patients may present with impaired extraocular movements, anisocoria, ptosis, proptosis, and decreased sensation in the forehead and upper eyelid. Immediate and accurate diagnosis is crucial, as delayed treatment can result in permanent nerve damage and loss of ocular function.

Management of SOFS often involves a multidisciplinary approach, including neurosurgery, maxillofacial surgery, and ophthalmology, to address the complex anatomical challenges and prevent long-term complications. Early surgical intervention is typically required to decompress the affected nerves and repair associated fractures. The timing of surgery is critical; prompt intervention has been shown to improve outcomes, while delays can lead to irreversible deficits.

In this report, we present the case of a 16-year-old male who developed complete ophthalmoplegia secondary to a SOF fracture following a motorized scooter accident. Despite initial conservative management, the patient’s condition deteriorated, necessitating surgical intervention. The successful decompression and reduction of the fracture led to a full recovery of ocular function, illustrating the importance of timely surgical management in such cases. This case underscores the need for heightened awareness of SOFS in patients presenting with orbital trauma and highlights the critical role of early surgical intervention in preventing permanent disability.

**Fig. 1:**
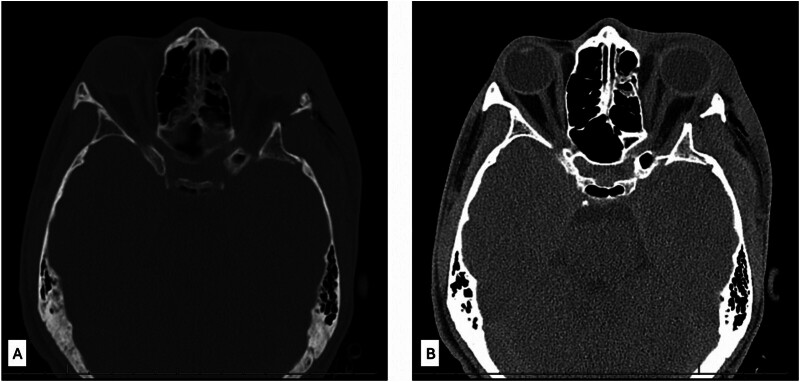
( **A** ) Computed tomography (CT) axial bone window showing fracture across the left greater wing of the sphenoid. ( **B** ) CT axial soft tissue window demonstrating impinging of the displaced left lateral wall in the lateral rectus muscle.

**Fig. 2:**
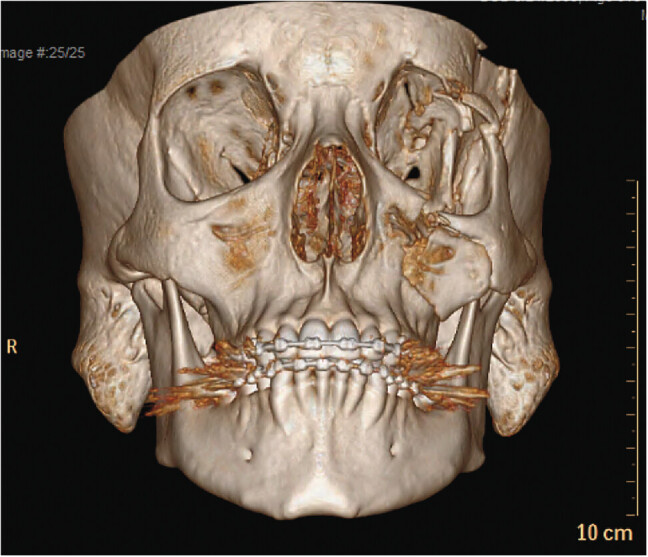
Computer-generated three-dimensional reconstruction of the facial bones.

Computed tomography (CT) scan reveals patient sustained significant head and facial injuries: (1) depressed left temporal skull fracture, (2) small left frontal epidural hematoma, (3) mild pneumocephalus, (4) left temporal epidural hematoma, (5) fracture of left sphenoid’s greater wing, (6) fractures of orbital floor, medial wall, and lateral wall of left orbit, (7) left zygoma fracture, (8) anterior and posterior left maxillary sinus fracture, (9) left frontal bone fracture, (10) left frontal sinus fracture ( Figs. 1 and 2 ). Neurosurgery and oral and maxillofacial surgery were consulted following these findings.

On hospital day 1, patient had a GCS 15. Visual acuity was at least counting fingers in both eyes. Intraocular pressure by tonometer was 15 mm Hg in the right eye and 18 mm Hg in the left eye, compared with 32 mm Hg a day prior. Both pupils were dilated, unreactive to light and accommodation. The right globe had normal motility. The left eye had diffused swelling with decreased motility to external exam. Both eyes had normal slit-lamp exam and vitreous was clear. Patient underwent CT angiogram of head and neck to evaluate the presence of any possible vascular injuries, which was normal. On hospital day 5, he started to develop complete ophthalmoplegia on the left eye with complete palsy of cranial nerve III, IV, and VI. He then underwent cerebral angiogram to rule out any possible carotid cavernous fistula that could potentially explain the reason for the ophthalmoplegia. On hospital day 7, patient developed anisocoria with dilated left pupil 6 mm, with marked deficit in all fields of gaze, compared with right pupil 2 mm. This is likely secondary to traumatic mydriasis with motility deficit, suspicious for involvement of cranial nerve III, IV, and VI. On the same day, patient was taken to the operating room for surgical decompression of SOF and reduction of the fractures. Delayed onset of ophthalmoplegia several days after craniofacial trauma has been described and is believed to result from evolving edema or progressive displacement of fracture fragments. 2 3

Intraoperative, initial stage involved performing a left frontotemporal craniotomy. Under the microscope, drilling of the greater wing of sphenoid bone was performed. Meningio-orbital band was identified, coagulated and cut. After gentle dissection and retraction of temporal lobe, no deformity was identified in foramen rotundum, foramen ovale, or foramen spinosum. Next, the SOF was found to be completely compressed by the displaced fracture of the sphenoid bone. At this stage, 3-mm diamond drill was used to decompress the entire SOF from any pressure points. Cranialization of the frontal sinus was performed by removing the tubular interna and entire sinus mucosa. At the last stage of the surgery, fractures of the lateral and superior orbital rim were reduced. Patient did well postoperatively here and eventually discharged from the hospital with complete recovery of his left eye function.

**Fig. 3:**
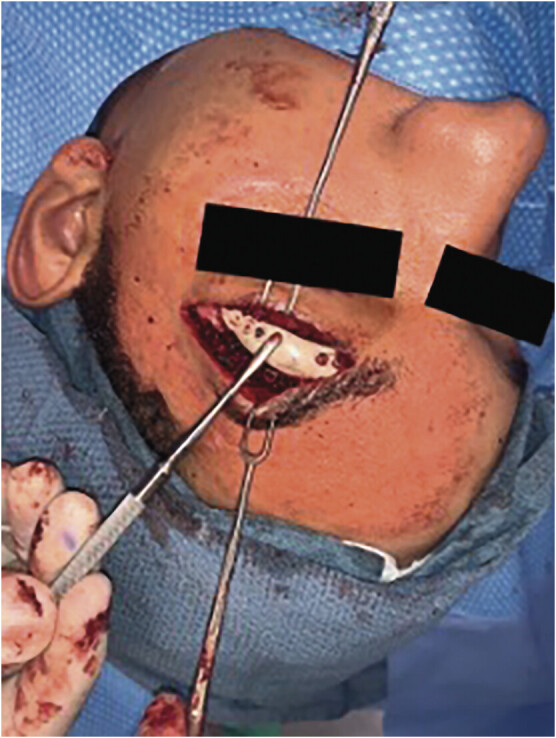
Custom implant based on imaging of the normal contralateral side.

Seven months postoperative, patient presents to the clinic to discuss reconstruction due to abnormal contour of his left brow region, which appears to be prominence laterally and flattening medially. The custom implant was designed preoperative based on imaging of the normal contralateral side. Intraoperatively, the left temporal plate was found to be malpositioned. It was then subsequently removed and replaced with the custom plant ( Fig. 3 ).

SOFS is a challenging condition that requires prompt identification and treatment. Although SOFS may be present at initial evaluation, several studies have reported delayed neurological deterioration, sometimes occurring 3 to 7 days after injury. 3 4 This pattern reinforces the need for serial ophthalmologic and cranial nerve examinations in patients with sphenoid or orbital apex fractures, even when early imaging or exam findings appear reassuring. In our patient, the initial examination showed only limited ocular motility, but the progression to complete ophthalmoplegia by hospital day 5 signaled worsening compression of cranial nerves III, IV, and VI. This emphasizes the need for serial examinations in patients with sphenoid or orbital ape fractures, as neurological decline may not be evident on presentation.

The timing of surgical decompression remains an area of debate. In the largest reported series of traumatic SOFS, early operative decompression within 7 to 10 days of symptom onset was associated with significantly higher rates of full cranial nerve recovery. 5 Once complete ophthalmoplegia develops, most authors advocate urgent surgical intervention to prevent irreversible axonal injury. 5 6 The present case supports this principle: decompression after neurological decline resulted in full functional recovery.

Similar reports of delayed traumatic SOFS describe variable outcomes ranging from partial improvement to full recovery, depending on the severity of compression and timing of surgery. 3 4 5 6 Our case adds to the literature by demonstrating complete neurological recovery despite delayed onset, followed by successful secondary reconstruction for cosmetic deformity.

Sphenoid wing fractures involving the SOF require vigilant monitoring due to the risk of delayed-onset SOFS. Once progressive ophthalmoplegia develops, urgent decompression is warranted to optimize neurological recovery. This case emphasizes the value of early recognition, multidisciplinary evaluation, and timely operative management.
